# Broadband and Wide‐Angle Nonreciprocal Electromagnetic Transport via Gradient Permeability‐Near‐Zero Metastructures

**DOI:** 10.1002/nap2.70112

**Published:** 2026-04-30

**Authors:** Junyang Sui, Hai‐Feng Zhang

**Affiliations:** ^1^ College of Electronic and Optical Engineering & College of Flexible Electronics (Future Technology) Nanjing University of Posts and Telecommunications Nanjing China; ^2^ State Key Laboratory of Millimeter Waves Southeast University Nanjing China

**Keywords:** broadband nonreciprocity, permeability‐near‐zero, robustness, transverse electric‐polarized leaky mode, wide‐angle, yttrium iron garnet

## Abstract

Breaking time‐reversal symmetry to achieve broadband and wide‐angle nonreciprocity is critical in electromagnetic waves manipulation. While Epsilon‐Near‐Zero materials have revolutionized the control of Transverse Magnetic modes, realizing comparable broadband nonreciprocity for Transverse Electric (TE) modes remains a formidable challenge because the permeability of the magneto‐optical materials is isotropic. Here, we propose a novel strategy utilizing gradient Permeability‐Near‐Zero (PNZ) subwavelength magnetic metastructures based on yttrium iron garnet thin films. By engineering the magnetic permeability tensor near the zero‐index regime, we excite robust TE‐polarized leaky modes (analogous to magnetic Berreman modes) in deep sub‐wavelength films (<*λ*/100, *λ* is wavelength). This mechanism unlocks a continuous nonreciprocal window, achieving a maximum nonreciprocity of 0.93 (approx. 12.4 dB) across the entire 35–40 GHz band. Unlike resonance‐dependent metastructures, our design exhibits intrinsic robustness, maintaining high performance over an exceptionally wide incidence angle range (> 40°) and thickness tolerances (−28% to +24%). This work fills the critical gap in magnetic light–matter interactions, offering a scalable and robust material platform for next‐generation directional electromagnetic energy transport, emission control, and signal isolation.

## Graphical abstract

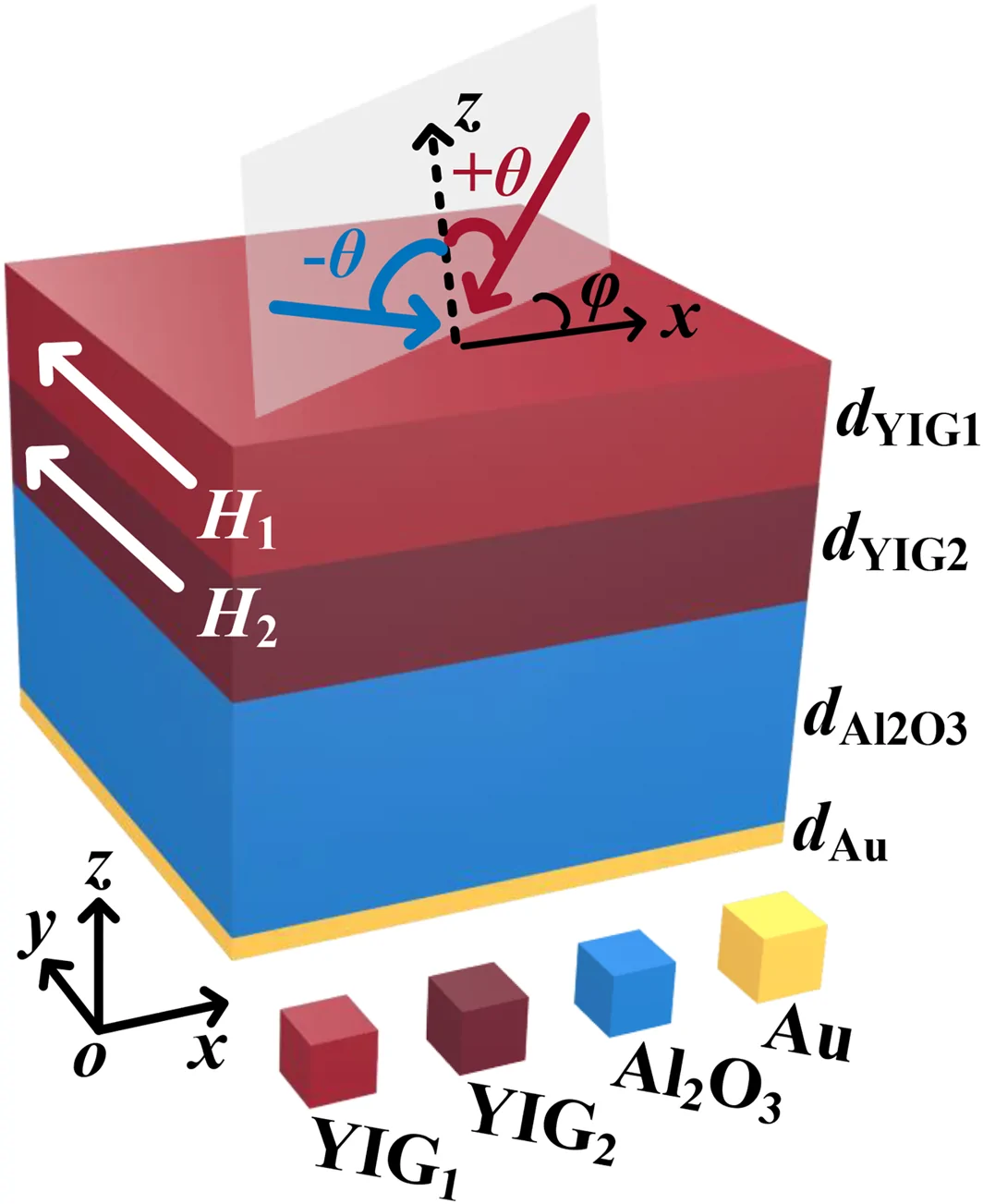

## 1 Introduction

The precise manipulation of amplitude [1], phase [2], and polarization [3] at subwavelength scales through metastructures, metamaterials, and metasurfaces has become the mainstream approach for functional control of electromagnetic (EM) waves. This has driven numerous applications, including perfect absorption [4], programmable transmission‐reflection structures [5], and precise polarization conversion [6]. However, compared to amplitude‐phase engineering [7] that addresses “how much” and “how” (reflection/transmission/absorption) to transmit, controlling the directionality of “where from”, particularly providing distinctly different scattering responses to EM waves propagating from different directions in free space, remains a frontier challenge with both theoretical and engineering significance [8]. Directionality selection and control are fundamental challenges in advanced nanophotonic and electromagnetic systems [9], directly impacting nonreciprocal optical signal isolation, directional energy harvesting, and asymmetric thermal emission control [10, 11, 12, 13]. For instance, in an ideal nonreciprocal thermal emitter or optical signal isolator, when electromagnetic waves from a target direction are incident at a positive angle (+*θ*) on one side of the normal, strong absorption is required to suppress backscattering and mutual interference. Conversely, when pointing to free space at a negative angle (−*θ*) on the opposite side, low loss and high reflection/transmission are necessary to preserve the efficiency of the outgoing emission [14]. Such direction‐selective nanophotonic and electromagnetic scenarios impose urgent requirements for broadband non‐reciprocity and wide‐angle tolerance [15, 16].

Existing directional devices primarily achieve “quasi‐directional selectivity” through mechanisms such as geometric phase [17], dynamic phase gradient [18], or anisotropic coupling [19]. However, they commonly face three major bottlenecks. (i) Narrow bandwidth and high sensitivity to incident angle, with significant performance degradation occurring away from the design point [17]. (ii) Under reciprocity constraints, it is challenging to establish intrinsically distinct channels for + *θ* and −*θ* incident waves [18]. (iii) Enhancing directional discrimination (nonreciprocity) often requires complex feed structures, increasing fabrication/integration complexity and losses [20]. To overcome these limitations, nonreciprocal approaches based on multi‐resonator superposition, time‐varying metasurfaces, and synthetic moving media have been proposed [21, 22, 23, 24, 25, 26]. However, simultaneously achieving broadband operation and wide‐angle performance remains challenging. For instance, Zhang et al. [21] designed a low‐biased magneto‐optical metasurface utilizing magnetic Kerr‐type dimers, which achieved a nonreciprocal isolation of over 10 dB across a bandwidth exceeding 3 GHz via multiple resonances, but its operation was restricted to fixed incident angles. On the contrary, Yang et al. [22] achieved ±64° wide‐angle nonreciprocal beam steering using a self‐biased magnetic metasurface based on lanthanum‐doped barium hexaferrite, yet this approach is constrained by its narrow‐band characteristics. To date, research on nonreciprocal subwavelength devices that simultaneously achieve broadband and wide‐angle free‐space scattering control remains notably absent [27].

We drew upon existing research in the mid‐infrared band (8–20 μm), where researchers successfully achieved broadband, wide‐angle transverse magnetic (TM) mode nonreciprocal thermal radiation by exploiting the epsilon‐near‐zero (ENZ) properties of InAs or Weyl semimetals [28, 29, 30, 31]. This achievement stems from the intrinsic nonreciprocity of the materials and the ENZ‐induced angle‐insensitive Berreman mode (BM), also known as the TM‐polarized leaky mode. However, restricted by intrinsic material attributes, these advances remain confined to the domain of thermal radiation and are challenging to translate directly to the millimeter wave band. Moreover, since the permeability of the magneto‐optical materials employed is isotropic, this mechanism is inherently incapable of extending to the nonreciprocal control of TE mode of EM waves [28, 29, 30, 31]. As quintessential magnetic materials in the microwave regime, ferrites exhibit gyrotropic anisotropy under an external magnetic bias. Their permeability tensor is characterized by $\vec{\mu }\left(\hat{y}Η\right)=\left[\mu_{r},0,-i\mu_{k};0,\mu_{0},0;i\mu_{k},0,\mu_{r}\right]$ [23, 32, 34], where the off‐diagonal elements break time‐reversal symmetry, serving as the fundamental basis for intrinsic nonreciprocity. Furthermore, the resonance frequency, phase shift, and impedance of ferrites can be continuously tuned by varying the magnetic bias strength and its applied direction [32]. Their intrinsic non‐reciprocity and tunability make ferrites frequently employed as magnetic materials for achieving non‐reciprocal properties in microwave devices such as isolators, circulators, and filters [32, 33]. More critically, our research reveals that when ferrites operate in the permeability‐near‐zero (PNZ) dispersion region, the main‐axis permeability component *μ*_*r*_→0 excites an angle‐insensitive matched dissipation channel under obliquely incident TE mode EM waves. The channel is known as the BM, also referred to as the TE‐polarized leaky mode (TPLM) [35]. This arises from the boundary condition requiring continuity of the normal component of magnetic flux density *B*. To maintain *B*_*z*_ = *μ*_*r*_ *H*_*z*_ finite while *μ*_*r*_ is extremely small, a large magnetic field component Hz must form within the material, given by $H_{z}=\frac{H_{i}\mu_{i}}{\mu_{r}\to 0}\to \infty$ (where *H*_*i*_ and *μ*_*i*_ represent the magnetic field and permeability of the adjacent layers). Combined with the Kramers‐Kronig relation, the imaginary part Im(*μ*_*r*_) of the permeability increases in this region, leading to rapid enhancement of magnetic losses and thus boosting EM waves absorption. Additionally, the increase in the ratio |*μ*_*k*_|/|*μ*_*r*_| between the main‐axis component and off‐diagonal components of permeability within the PNZ amplifies the nonreciprocal effect. Leveraging this physical mechanism and the tunability of ferrites, the continuous PNZ region provides a theoretical foundation for achieving broadband and wide‐angle nonreciprocal absorption/reflection in free space. This also establishes the basis for subsequent structural design and mechanism development in the operation frequency range discussed in this paper.

To address these demands and bridge the existing research gap, this work designs subwavelength magnetic metastructures based on gradient PNZ Yttrium Iron Garnet (YIG) films, realizing robust broadband and wide‐angle nonreciprocal absorption/reflection as a macroscopic physical model in the operation band. Firstly, we investigate the critical role of PNZ characteristics and dispersion relations of magnetized YIG in enabling strong nonreciprocal absorption within deep subwavelength YIG films (<*λ*/100, *λ* being operation wavelength) under moderate external magnetic field strength *H*. This approach effectively overcomes the performance limitations of conventional reciprocal and nonreciprocal systems (see Figure 1a). Subsequently, building upon this physical mechanism, the Artificial Rabbits Optimization algorithm is employed to optimize a dual‐layer YIG/dielectric/Au metastructures. By tailoring the magnetic field *H*, the PNZ resonance frequencies of the two YIG layers are staggered to form a gradient response, covering the range of 35–40 GHz. Broadband high absorption is achieved by exciting the TPLM via the enhanced *z*‐component *H*_*z*_ of the magnetic field of the incident TE mode of EM wave, while the intrinsic nonreciprocity of the YIG ensures asymmetric absorption/reflection. Finally, we systematically evaluate the impact of the incidence angle *θ*, azimuth angle *φ*, dielectric material performance, and thickness tolerances on the broadband nonreciprocal performance. This work provides a novel design strategy for directional scattering and nonreciprocal light–matter interaction. Furthermore, while validated in the microwave regime, this macroscopic gradient PNZ platform establishes a highly translatable theoretical framework for designing next‐generation directional nanophotonic devices, robust thermal emitters, and signal isolators in the terahertz and infrared bands.

**FIGURE 1:**
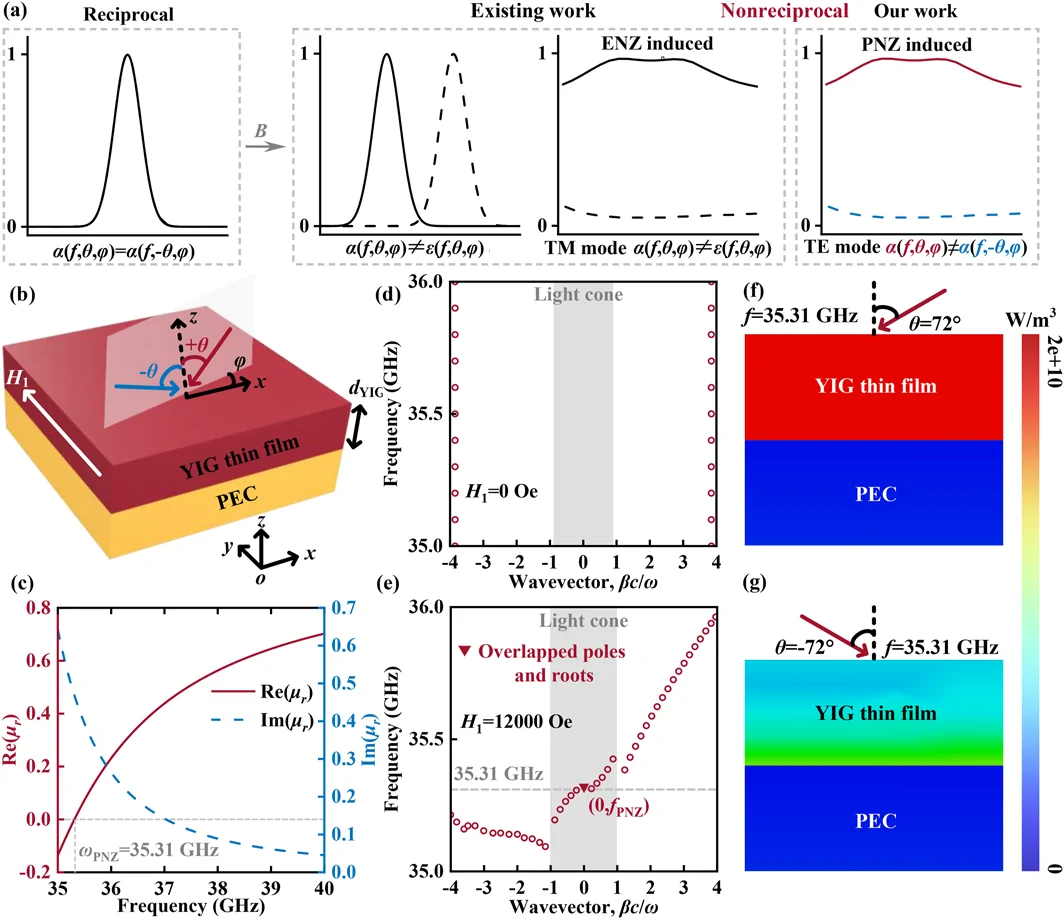
Schematics of nonreciprocal absorption control. (a) Compared absorption spectra of reciprocal work (left gray dashed frame), existing nonreciprocal thermal radiation work (middle gray dashed frame) and our nonreciprocal absorption work (right gray dashed frame). The overlapped spectra are separated in the presence of *B*. (b) Schematic of a single magnetized YIG thin film (thickness *d*_YIG_ = 65 μm) atop the PEC substrate. The external magnetic field *H*_1_ is applied along the +*y*‐axis direction. Here, *θ* is the incident angle and *φ* is the azimuth angle. (c) Permeability of the YIG film at *H*_1_ = 12000 Oe. Dispersion relation of the YIG and PEC configuration in the lossless limit, under different values of *H*_1_: (d) 0 Oe, and (e) 12000 Oe. Distribution of EM power dissipation density within the configuration at *f* = 35.31 GHz, with *θ* set to (f) 72°, and (g) −72°, respectively.

## 2 Physical Principle and Configuration

As illustrated in Figure 1b, we first consider an opaque and nonreciprocal configuration consisting of a thin YIG film (its thickness is *d*_YIG_) loaded atop a perfect electric conductor (PEC) substrate. The white rectangle represents the plane of incidence, which forms an azimuthal angle *φ* with the +*x*‐axis. The red and blue arrows denote EM waves incident at angles of +*θ* and −*θ* within this plane, respectively. An external magnetic field *H*_1_ is applied along the +*y*‐axis, inducing gyrotropic anisotropy in the YIG film [33, 34] (calculations of the YIG EM properties are provided in Section 1 of Supporting Information S1). Figure 1c presents the relative permeability *μ*_*r*_ spectrum of the YIG film, calculated with a damping constant *Γ* = 0 and a saturation magnetization *M*_*S*_ = 1520 Oe. It demonstrates that the PNZ frequency is located at 35.31 GHz under a bias field of *H*_1_ = 12000 Oe. Such a thin PNZ film backed by a PEC substrate supports guided modes with TE polarization (*H*_*x*_,*E*_*y*_,*k*_*z*_), whose dispersion relation is governed by

(1) $$\tan\left(\kappa d_{\text{YIG}}\right)=\frac{\mu_{r}\kappa }{\mu_{k}\beta -\left(\mu_{r}^{2}-\mu_{k}^{2}\right)}$$

where *β* represents the propagation constant, $\kappa =\sqrt{k_{0}\varepsilon_{f}\frac{\mu_{r}^{2}-\mu_{k}^{2}}{\mu_{r}}-\beta^{2}}$ (*k*_0_ = *ω*/*c* = 2π/*λ*, *ω* and *c* are angular frequency and the speed of light in a vacuum, respectively). Also, *ε*_*f*_ = 15 is the permittivity of the YIG [33, 34]. A detailed derivation of the dispersion relation is provided in Section 2 of Supporting Information S1. Figure 1d,e illustrate the dispersion curves for a 65 μm YIG film (*d*_YIG_ < *λ*/100) backed by a PEC substrate, calculated in the lossless limit. The radiative BM, first investigated by Berreman in 1963 [35], resides within the light cone and can directly couple with free‐space EM propagation modes, inducing resonant absorption peaks in the far‐field spectrum [36]. Generally, in the absence of an applied magnetic field (*H*_1_ = 0 Oe), the entire system is reciprocal, exhibiting a fully symmetric dispersion profile where *ω*(‐*β*) = *ω*(*β*), as shown in Figure 1d. However, in the presence of a bias field of *H*_1_ = 12000 Oe, the dispersion curves within the light cone undergo a rotation relative to the center point (0, *f*_PNZ_) (see Figure 1e). This center of rotation corresponds to a bound state in the continuous spectrum at the lossless limit [37], a state where the roots and poles of Equation (1) perfectly overlap [38]. For symmetric wave vectors *β*(±*θ*), the application of *H*_1_ causes the mode frequencies to split and shift in opposite directions. Consequently, this symmetry‐breaking behavior provides a novel platform for tailoring nonreciprocal absorption and reflection. In the presence of an opaque substrate, the absorptivity and reflectivity are related by

(2) α(f,θ,φ)=1−R(f,θ,φ)

The relationship is calculated using the transfer matrix method (TMM) [33, 34] as detailed in Section 3 of Supporting Information S1. To elucidate the physical mechanism governing the nonreciprocal absorption under ±*θ* incidence within the PNZ regime, we visualize the EM power dissipation density (denoted as *Q*_diss_) of the structure (Figure 1b) at the PNZ frequency (*f* = 35.31 GHz), as shown in Figure 1f,g. Here, *Q*_diss_(*x*, *y*, *z*)|(*f*, *θ*, *φ*) denotes the spatial power density of absorption/dissipation of the structure at specific frequencies *f*, angles *θ*, and phases *φ*, which relates to *α*(*f*, *θ*, *φ*) through the simple relationship

(3) $$\alpha \left(f,\theta ,\varphi \right)=\frac{\int_{V\left(x,y,z\right)}Q_{\text{diss}}\left(x,y,z\right){|}_{f,\theta ,\varphi }dV}{P_{\text{incident}}{|}_{f,\theta ,\varphi }}$$

When the incident angle *θ* = 72°, the dissipation density *Q*_diss_ is localized within the YIG thin film (see Figure 1f), indicating that the *H*_*z*_ magnetic field component of the EM waves is confined within them due to TPLM. Furthermore, the presence of magnetic losses Im(*μ*_*r*_) (shown in Figure 1c) leads to high absorption. Conversely, at *θ* = −72°, the symmetry‐breaking scattering response induced by the magnetic bias prevents the propagation of corresponding EM wave modes within the material. Consequently, the intensity of *Q*_diss_ in the YIG film diminishes drastically, as shown in Figure 1g. This corresponds to a significant reduction in absorptivity *α*(*f*, *θ*, *φ*), which, according to Equation (2), translates to the generation of high reflectivity *R*(*f*, *θ*, *φ*).

Building upon the theoretical platform discussed above, Figure 2 illustrates the subwavelength magnetic metastructures designed to achieve broadband nonreciprocal absorption and reflection within the target frequency band (35–40 GHz). Distinct materials are filled with different colors for clarity. The stack comprises two top ferrite layers (denoted as YIG_1_ and YIG_2_) with distinct Γ and *M*_*S*_, an intermediate dielectric layer of Al_2_O_3_ (kept constant unless otherwise specified), and a bottom Au substrate. The layer thicknesses are optimized as *d*_YIG1_ = 65 μm, *d*_YIG2_ = 50 μm, *d*_Al2O3_ = 480 μm, and *d*_Au_ = 1 μm. The permittivity of Al_2_O_3_ is taken as 9.8 [39]. The optical properties of Au follow the Lorentz‐Drude model [40]. The thickness of Au layer exceeds the skin depth and in the operation band, it effectively functions as a PEC with zero transmissivity, thereby ensuring the validity of Equation (2). External magnetic fields *H*_1_ = 12000 Oe and *H*_2_ = 12900 Oe are applied along the +*y*‐axis to the YIG_1_ and YIG_2_ layers, respectively. A feasible experimental process for the proposed metastructure is shown in Supporting Information S1: Section 4. Their magnitudes are tuned to enable the PNZ frequencies of the two YIG layers to cover the 35–40 GHz range. Detailed parameters for YIG_1_ and YIG_2_ are listed in Table 1. For convenience in subsequent performance evaluation, we also directly provide the formulas for the average absorptivity *α*_average_(*f*, *θ*, *φ*), nonreciprocity *η*(*f*, *θ*, *φ*), and average nonreciprocity *η*_average_(*f*, *θ*, *φ*) as follows [41, 42].

(4) $$\alpha_{\text{average}}\left(f,\theta ,\varphi \right)=\frac{\int_{f_{L}}^{f_{H}}\alpha \left(f,\theta ,\varphi \right)df}{f_{H}-f_{L}}$$

(5) η(f,θ,φ)=|α(f,θ,φ)−α(f,−θ,φ)|

(6) $$\eta_{\text{average}}\left(f,\theta ,\varphi \right)=\left|\alpha_{\text{average}}\left(f,\theta ,\varphi \right)-\alpha_{\text{average}}\left(f,-\theta ,\varphi \right)\right|$$

**FIGURE 2:**
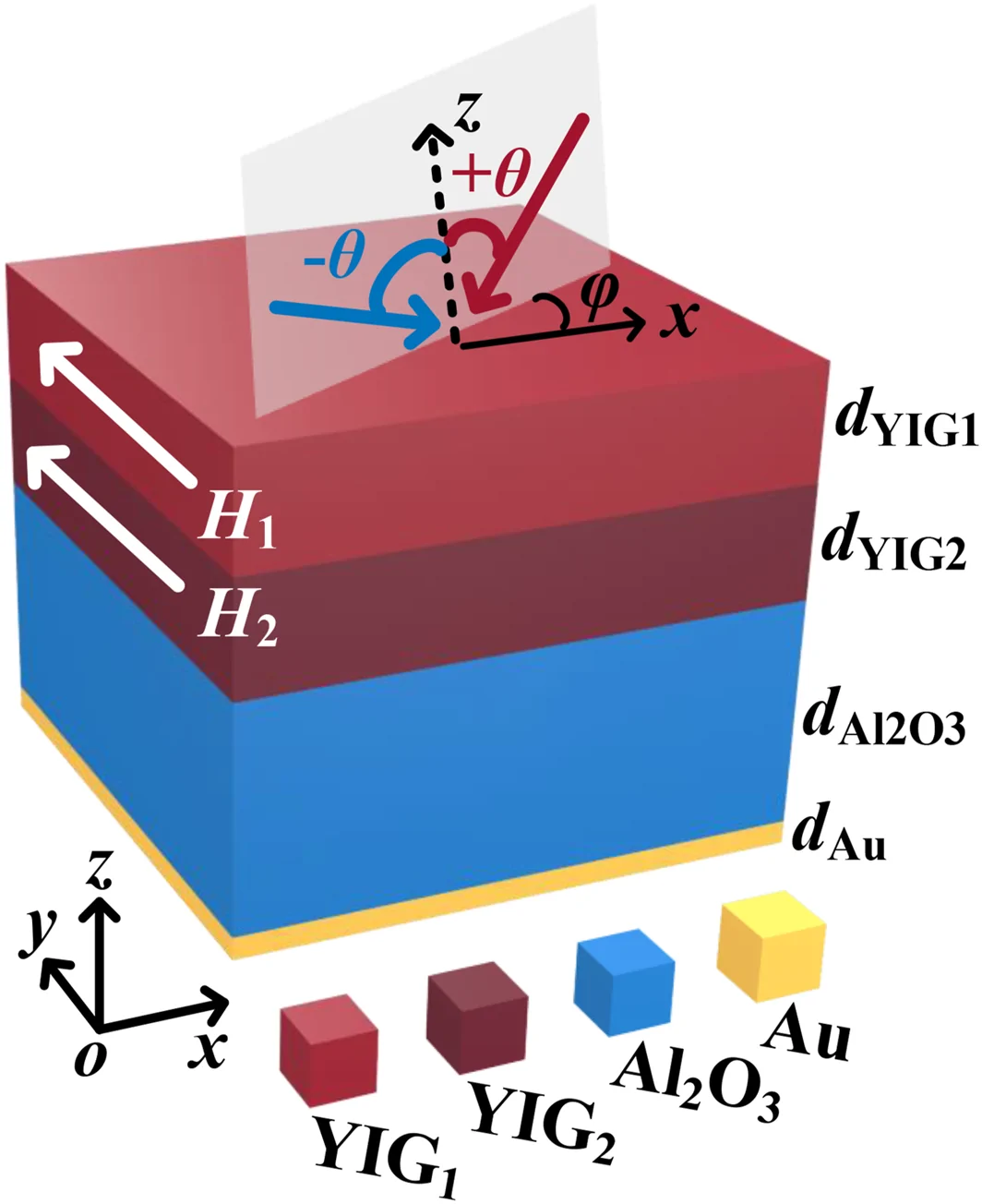
A schematic illustration of the dual‐layer YIG/Al_2_O_3_/Au metastructures consisting of YIG_1_ and YIG_2_ with different Γ and *M*_*S*_. The white box is the incident plane at an azimuth angle *φ* between the + *x*‐axis, TE mode EM waves incident at + *θ* (red line) and ‐*θ* (blue line). The magnetic fields *H*_1_ and *H*_2_ are applied parallel to the + *y*‐axis.

**TABLE 1:** The properties parameters of YIG_1_ and YIG_2_.

Layer	Γ	*M*_*S*_ (Oe)
YIG_1_	0.022	1520
YIG_2_	0.025	1800

All parameters constituting the metastructures mentioned above are optimized using a Artificial Rabbits Optimization algorithm to maximize nonreciprocal performance (see Supporting Information S1 Section 5 for the detailed optimization process) [43, 44].

## 3 Analysis and Discussion of Performance

The fundamental distinction between reciprocal and non‐reciprocal scattering responses corresponding to (*f*, θ, *φ*) and (*f*, *θ*, *φ*) is illustrated in Figure 1a. Reciprocal systems preclude directional selectivity in the spectrum, keeping to *α*(*f*, *θ*, *φ*) = *α*(*f*, −*θ*, *φ*) [18]. While nonreciprocal systems incorporating magneto‐optical materials can break this symmetry, the spectral difference between ±*θ* achieved via conventional techniques, such as guided mode resonances or Fabry‐Pérot (FP) resonances, is typically constrained to narrow bandwidths [17, 41, 42]. Existing studies have utilized gradient infrared ENZ materials, such as InAs or Weyl semimetals, to realize broadband nonreciprocal absorption. However, constrained by intrinsic material attributes, these approaches are strictly limited to the TM mode and cannot be directly translated to the microwave frequency band. To solve these challenges, the metastructures designed in Figure 2 achieve gradient PNZ coverage by adjusting the YIG layers via magnetic field *H*, thereby generating broadband nonreciprocity in TE mode. For simplicity, we first focus on the calculation for the incident plane at *φ* = 0°. Figure 2a shows the absorption spectrum at *θ* = ± 72° obtained using the artificial rabbits optimization algorithm. Within the 35–40 GHz frequency range, *α*(*f*, 72°, 0°) > 0.85 and *α*(*f*, −72°, 0°) < 0.12 are consistently satisfied, with the nonreciprocity *η*(*f*, 72°, 0°) peaking at 0.93. Using Equation (4), the calculated average absorptivity are *α*_average_(*f*, 72°, 0°) = 0.94 and *α*_average_(*f*, −72°, 0°) = 0.09. These results demonstrate that the designed metastructures exhibit significant directional absorption selectivity. According to Equation (2), this implies simultaneous directional reflection selectivity in the opposite direction.

We observe that distinct absorption peaks are at two identical frequency points (marked by triangles in Figure 3a) when *θ* = ± 72°, occurring at 35.66 and 38.55 GHz. Section 6 of Supporting Information S1 verifies the accuracy of the calculation results and absorptivity spectra under TM mode. To provide clearer physical insight into the absorption peaks, Figure 3b displays the real part (Re(*μ*_*r*_)) and imaginary part (Im(*μ*_*r*_)) of the main‐axis permeability. The zero‐crossing of Re(*μ*_*r*_) at specific frequencies of 35.31 and 38.1 GHz for the two ferrite layers YIG_1_ and YIG_2_ also reveals the blue shift behavior of the PNZ with increasing magnetic field strength *H*. The PNZ YIG thin films exhibit outstanding performance in magnetic field energy localization for EM waves, attributed to boundary conditions $H_{\text{PNZ}}=\frac{H_{i}\mu_{i}}{\mu_{r}\to 0}\to \infty$ satisfying continuous normal magnetic flux density *B*. The presence of magnetic loss Im(*μ*_*r*_) contributes to energy absorption. Consequently, near the PNZ, BM, or TPLM is excited. These modes are crucial for coupling free‐space EM waves into the material at an oblique angle *θ*. The coupling frequencies can be analyzed using the magnetic energy loss function, denoted by Im(−1/*μ*_*r*_), analogous to the approach used for ENZ materials [28, 29, 30, 31]. This function exhibits peaks near the PNZ points, specifically at 35.66 and 38.55 GHz for YIG_1_ and YIG_2_, respectively. Therefore, the stacked PNZ films facilitate broadband directional absorption across the range of 35–40 GHz. Figure 3c illustrates the ratio of the off‐diagonal component |*μ*_*k*_| to the diagonal component |*μ*_*r*_| of the YIG permeability tensor within the 35–40 GHz operating band. As expected, two distinct peaks are observed near the PNZ frequencies of the two YIG layers. Due to the condition, *μ*_*r*_→0, the magneto‐optical strength, characterized by the Voigt parameter $\zeta =\frac{\left|\mu_{k}\right|}{\left|\mu_{r}\right|\to 0}\to \infty$, is significantly enhanced regardless of how small |*μ*_*k*_| is, indicating strong nonreciprocity. In terms of experimental feasibility, different external magnetic fields can be applied to the respective YIG layers utilizing gradient magnetic field coils. By controlling the spatial distribution of applied currents, a linear magnetic field gradient can be generated [45], satisfying the requirement for distinct localized field intensities (*H*_1_≠*H*_2_). This gradient is essential for the broadband response, as further discussed in the Section 7 of Supporting Information S1, applying a uniform magnetic field would cause the PNZ resonance peaks of the two YIG layers to overlap, significantly narrowing the operational bandwidth.

**FIGURE 3:**
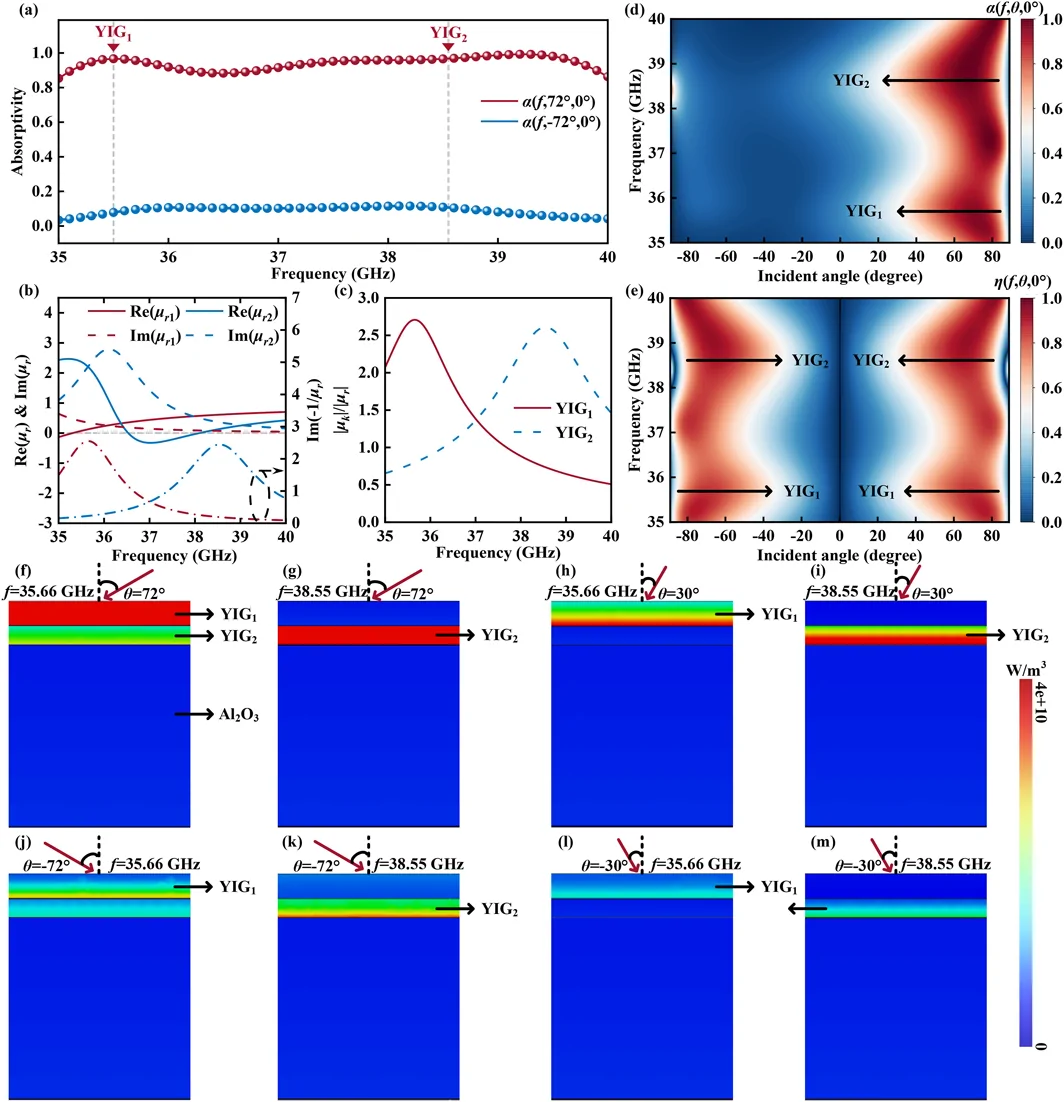
Performance and analysis of the nonreciprocal metastructures. (a) Absorptivity spectra at *θ* = ± 72° show distinct separation. Corresponding PNZ resonance absorption peaks for each YIG layer are marked with triangles. (b) Permeability tensor curves for YIG_1_ and YIG_2_. The solid and dashed lines represent the real and imaginary parts *μ*_*r*_ of YIG materials, respectively. The dash‐dotted lines indicate the frequencies of TPLM obtained from the energy‐loss function Im(−1/*μ*_*r*_). (c) The ratio of permeability components |*μ*_*k*_| to |*μ*_*r*_| within the frequency range of 35–40 GHz for the two YIG layers. The two peaks around 35.66 and 38.55 GHz correspond to the largest nonreciprocity in this frequency range, attributed to the PNZ properties. (d) Contour map of *α*(*f*, *θ*, 0°) of the metastructures across a wide frequency range from 35 to 40 GHz at different *θ*. (e) Contour map displaying the *η*(*f*, *θ*, 0°) between *α*(*f*, *θ*, 0°) and *α*(*f*, −*θ*, 0°). (f)–(m) Distribution of EM power dissipation density within the metastructures at the corresponding frequency, with *θ* set to ± 72° and 30°, respectively.

The contour plot of *α*(*f*, *θ*, 0°) for the designed metastructures are presented in Figure 3d, spanning a frequency range of 35–40 GHz and incidence angles from −89° to 89°. The absorptivity exhibits pronounced asymmetry across the entire angle spectrum. High absorptivity exceeding 0.6 is observed over a wide angle range and throughout the ultra‐wide operational band. Notably, within the frequency bands where the TPLM is excited by the PNZ properties of YIG_1_ and YIG_2_, the high‐absorptivity window (*α*(*f*, *θ*, 0°) > 0.6) extends over a broader angle range (black arrows) compared to other frequencies, underscoring the angler robustness inherent to the PNZ behavior. Furthermore, the intrinsic material loss of the two YIG layers contributes to maintaining high absorption in bands outside the PNZ region. Given that the TPLM in YIG films is predominantly excited at large angles (where the *H*_*z*_ component is maximized), the incorporation of the embedded Al_2_O_3_ dielectric layer in the given design appears to effectively mitigate the degradation of absorptivity at smaller angles. In contrast to previous gradient ENZ studies utilizing bare stacks of InAs or Weyl semimetals (without dielectric interlayers), which achieved broadband high absorption only at specific angles which have large values [28, 29, 46], we attribute the enhanced absorption at near‐normal incidence in metastructures primarily to the presence of the dielectric layer. This layer effectively introduces an additional optical cavity between the YIG stack and the bottom Au substrate. This configuration promotes multiple internal reflections of EM waves within the cavity, thereby enhancing the absorption within the top YIG bilayers. Consequently, the dielectric layer must exhibit minimal loss within the 35–40 GHz band, justifying the selection of Al_2_O_3_. The function of the cavity can be referenced from previous studies utilizing cavities for FP resonance to absorb EM waves [47].

Figure 3e shows the difference *η*(*f*, *θ*, 0°) between the absorptivity *α*(*f*, *θ*, 0°) and *α*(*f*, −*θ*, 0°) at the polar angles on either side of the normal to the metastructures, where a larger *η*(*f*, *θ*, 0°) indicates stronger nonreciprocity. Since it represents |*α*(*f*, *θ*, 0°)−*α*(*f*, −*θ*, 0°)|, the contour plot of *η*(*f*, *θ*, 0°) exhibits symmetry relative to the normal direction. A significant contrast between the absorptivity at ±*θ* is observed across the broad frequency band of 35–40 GHz within the angle range of 0°–89°. This contrast is particularly pronounced at large oblique angles, for instance, from 58° to 88°, which is attributed to the stronger contribution of the TPLM in this region. Although the nonreciprocity diminishes as *θ* approaches zero, a notable difference (*η*(*f*, *θ*, 0°) > 0.1) persists within the range of *θ* = 5°–20°. This indicates that the robust nonreciprocal absorption, generated by the dielectric film between YIG and Au layers, can extend even to smaller angles.

We visualize the EM power dissipation density *Q*_diss_ distributions of the metastructures at varying *θ* and frequencies (35.66 and 38.55 GHz, marked by triangles in Figure 3a) using the Finite Element Method (FEM) [48], as shown in Figure 3f–m. Taking *θ* = 72° as a representative case, the power dissipation at 35.66 GHz is governed by two factors (see Figure 3f). The primary contribution arises from the absorption in the top YIG_1_ layer, driven by the excitation of the TPLM in the spectral vicinity of its PNZ frequency (35.31 GHz). Simultaneously, the underlying YIG_2_ layer also contributes to the power absorption owing to its inevitable intrinsic material loss. At the distinct frequency of 38.55 GHz (as presented in Figure 3g), the TPLM supported by YIG_2_ facilitates coupling with free‐space EM waves, resulting in the confinement and absorption of the majority of EM power near its PNZ frequency. For comparison, the *Q*_diss_ profiles at the same frequencies but under negative incidence ‐*θ* are plotted in Figure 3j,k. The TPLM in the dual‐layer YIG enhances non‐reciprocity, leading to a significant weakening of absorption at *θ* = −72°. This weaker absorption results in a much lower spectral value for *α*(*f*, −72°, 0°) in Figure 4a. A similar pattern is observed in the *Q*_diss_ distribution at other incidence angles, such as *θ* = 30° and −30° (see Figure 3h–m), where the TPLM effect gradually diminishes as *θ* approaches zero. Nevertheless, nonreciprocal absorption remains effective at these small angles due to the presence of the dielectric layer, which effectively introduces an additional cavity, thereby enhancing absorption at small angles.

**FIGURE 4:**
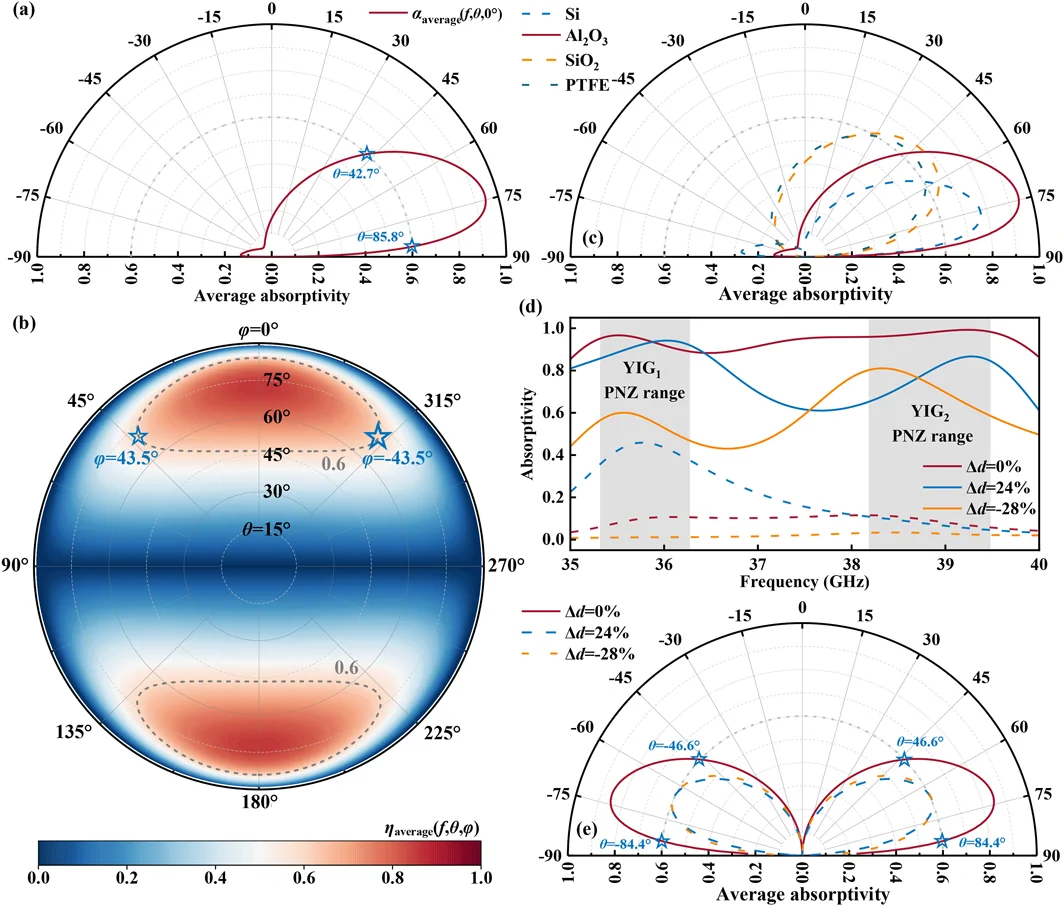
Schematics of the metastructures design stability and robustness analysis. (a) The average absorptivity *α*_average_(*f*, *θ*, *φ*) with *φ* = 0° across a broad frequency range from 35 to 40 GHz in *θ* polar coordinates from −90° to 90°. (b) Contour maps of frequency‐averaged nonreciprocity *η*_average_(*f*, *θ*, *φ*) of the metastructures in incidence plane with azimuth angle *φ* range from 0° to 360°. The radial direction represents the polar angle *θ* from 0° to 89°. (c) Corresponding average *α*_average_(*f*, *θ*, *φ*) of the metastructures with the dielectric layer material is Si, Al_2_O_3_, SiO_2_, and PTFE. (d) The absorptivity spectra at *θ* = ± 72° belong to thickness errors Δ*d* = 0%, 24%, and −28%. The gray shaded areas represent the PNZ regions of YIG_1_ and YIG_2_. (e) The *η*_average_(*f*, *θ*, *φ*) curves of Δ*d* = 0%, 24%, and −28% in *θ* polar coordinates.

For the TE mode, the averaged absorptivity is investigated over a wide frequency ranges 35–40 GHz (Figure 4a). The *α*_average_(*f*, *θ*, *φ*) curve exhibits significant asymmetry at *θ* with opposite signs. Leveraging the angle independence of PNZs, *α*_average_(*f*, *θ*, *φ*) exceeds 0.6 within a *θ* range of 43.1 (*θ* = 42.7°–85.8°). In contrast, absorption in the other half‐plane remains relatively low, consistently below 0.1. This demonstrates the wide‐angle nonreciprocal absorption advantage of the proposed metastructures. We further calculated the band‐averaged nonreciprocity *η*_average_(*f*, *θ*, *φ*) corresponding to different azimuth angles *φ* on the incident plane, sweeping *φ* from 0° to 360° and *θ* from 0° to 89° (as plotted in Figure 4b). Polarization conversion is also incorporated into the reflectance calculation (denoted as *R*_*ps*_(*f*, *θ*, *φ*), representing the conversion of incident TE mode waves into TM mode reflected waves), modifying the equation to *α*(*f*, *θ*, *φ*) = 1−*R*_*ss*_(*f*, *θ*, *φ*)−*R*_*ps*_(*f*, *θ*, *φ*). The off‐diagonal term *μ*_*k*_ of the YIG permeability tensor couples the *x*‐axis component *H*_*x*_ and *z*‐axis component *H*_*z*_ of the TE mode EM waves' magnetic field, exhibiting asymmetric effects at ±*θ*. Therefore, the nonreciprocal effect is strongest at *φ* = 0° and 180°, achieving *η*_average_(*f*, *θ*, *φ*) > 0.6 within a *θ* range of 37.8 (*θ* = 46.6°–84.4°). As *φ* approaches 90° and 270°, the *H*_*y*_ and *H*_*z*_ components of the TE mode EM waves dominate, while the coupling contribution from the *H*_*x*_ component diminishes (disappearing entirely at *φ* = 90° and 270°). This weakened coupling effect reduces the asymmetry, causing the nonreciprocity to gradually decrease toward zero. Although the *H*_*x*_ component weakens at incident angles other than 0° and 180°, the nonreciprocity remains robust over a substantial *φ* range of 97° (*φ* = −43.5°–43.5°).

From a practical standpoint, the use of alternative materials for the dielectric layer is discussed. Figure 4c displays the *α*_average_(*f*, *θ*, *φ*) for dielectric layers made of Si, Al_2_O_3_, SiO_2_, and Polytetrafluoroethylene (PTFE), arranged in descending order of permittivity. The permittivity data for these materials are obtained from previous experimental results [39, 49, 50]. The results demonstrate that metastructures can exhibit the desired nonreciprocal absorption properties regardless of the dielectric's optical characteristics. The observed significant nonreciprocity of the TE mode remains consistent over a broad angle range and wide bandwidth. Among the tested materials, Al_2_O_3_ exhibits the highest absorptivity within the *θ* range of 0°–89°. This correlates with the cavity thickness of 480 μm in Figure 2, which approximately satisfies the cavity resonance condition. Furthermore, Al_2_O_3_ has exceptionally low‐loss characteristics in the microwave regime [39]. Within the target 35–40 GHz band, Al_2_O_3_ exhibits negligible material dispersion, with its refractive index varying strictly within an order of 10^−3^. Consequently, its relative permittivity can be accurately treated as a constant value of 9.8 in our calculations. The critical role of this dielectric layer's loss in optimizing the phase‐matching conditions and overall nonreciprocal performance is further analyzed in Section 7 of the Supporting Information S1. It is also found that as the permittivity of the material in the dielectric layer gradually increases, the high average absorptivity shifts toward larger angles. Beyond the cavity thickness factor, a higher permittivity within the cavity also enhances the equivalent optical path length, which is more favorable for balancing the effects of large‐angle incidence.

We also investigate the sensitivity of nonreciprocal properties to manufacturing errors in metastructures, focusing particularly on thickness deviations in the two YIG layers. Δ*d* is defined as the thickness variation applied to the original dimensions *d*_YIG1_ = 65 μm and *d*_YIG2_ = 50 μm, following the formula *d*_total_ = *d*_origin_(1 + Δ*d*). At *φ* = 0°, Figure 4d displays the absorptivity spectra at *θ* = ± 72° for Δ*d* = 0%, 24%, and −28%. Although these thickness variations far exceed existing manufacturing tolerances such as Precision Lapping (Δ*d* = ± 5%) [51] and Liquid Phase Epitaxy (Δ*d* = ± 1%) [52], the absorptivity spectra retain TPLM presence within the PNZ regions of YIG_1_ and YIG_2_ due to YIG's thickness‐independent PNZ behavior. This manifests as nonreciprocal absorption peaks and maintains excellent nonreciprocity across the entire 35–40 GHz frequency band. Figure 4e shows the variation of the average nonreciprocity *η*_average_(*f*, *θ*, *φ*) with *θ*. Critical values of *η*_average_(*f*, *θ*, *φ*) exceeding 0.6 occur at Δ*d* = 24% and −28%. This indicates that broadband nonreciprocity of metastructures is stably achieved within the Δ*d* range of −28%–24%. This insensitivity to structural errors is fundamentally related to the physical mechanism of TPLM supported by PNZ YIG. Unlike geometric cavity resonances (e.g., FP resonant modes), which require strict phase‐matching conditions dependent on precise cavity length, TPLM is a radiation mode excited at the interface of PNZ materials. As long as the PNZ gradient distribution remains largely unchanged, the device exhibits a broader angular applicability and lower sensitivity to stacked film thickness. The Section 8 of Supporting Information S1 shows the discussion of YIGs' damping coefficient error (*η*_average_(*θ =* 72°) > 0.6 in the range of ΔΓ = −67%–422%) and saturation magnetization error (*η*_average_(*θ =* 72°) > 0.6 in the range of Δ*M*_*S*_ = −52%–73%). Additionally, loss tangent belongs to Al_2_O_3_ (*η*_average_(*θ =* 72*°*) > 0.6 in the range of tan*δ* = 0–0.0146) is also discussed in the Supporting Information S1: Section 8. Consequently, the proposed metastructures exhibit exceptional angle tolerance and resilience to fabrication errors, making them well‐suited for complex application scenarios and scalable mass production.

Table 2 presents a comparative analysis between this work and previously reported nonreciprocal metastructures. Prior studies have predominantly focused on optimizing individual performance metrics, such as maximizing the nonreciprocity or extending the angle range. However, such approaches often limit their applicability in scenarios requiring complex and multifunctional capabilities. In contrast, leveraging the novel mechanism of gradient PNZ YIG, the proposed TPLM achieves superior performance across four critical dimensions, that is, (i) a wider operating bandwidth (35–40 GHz), (ii) the higher nonreciprocity (*η*(*f*, *θ*, *φ*) = 0.93), (iii) the wider angle tolerance (*θ* = 42.7°–85.8° and *φ* = −43.5°–43.5°), and (iv) exceptional manufacturing robustness (Δ*d* = −24%–28%). Consequently, this work aims to provide a robust design strategy for broadband and wide‐angle nonreciprocal absorbers/reflectors, facilitating directional EM waves propagation and shielding in multifunctional environments.

**TABLE 2:** Comparison of nonreciprocal performance based on subwavelength metastructures.

Refs.	Years	Physical principles	Frequency range	Nonreciprocity	Angle range
[53]	2022	Unilateral power amplifier	5 GHz	25 dB	0°–20°
[22]	2023	Self‐biased magnetic meta‐atoms	13.7 GHz, 15.7 GHz	7 dB	0°–64°
[54]	2023	Spatio‐temporal modulation	2.4 GHz	11 dB	45°
[21]	2024	Multiple resonances	16.1–19.4 GHz	10 dB	0°
[55]	2025	YIG impedance matching	9–12 GHz	25 dB	0°
[32]	2025	YIG and Mie resonance	6.5–9 GHz	28.35 dB	0°–50°
Our work	2026	PNZ YIG	35–40 GHz	0.93 or 12.4 dB	42.7°–85.8°

## 4 Conclusion

In summary, we have proposed and demonstrated a robust material strategy for achieving broadband, wide‐angle nonreciprocal propagation of TE modes, effectively overcoming the limitations of conventional magnetic metastructures. By leveraging the gradient PNZ effect in magnetized YIG multilayers, we successfully excited TE‐polarized leaky modes that facilitate strong, direction‐selective absorption across the entire operation band (35–40 GHz). Our results reveal that the PNZ‐based mechanism fundamentally differs from geometric cavity resonances, offering superior angular stability (*θ* from 42.7° to 85.8°) and exceptional tolerance to fabrication imperfections (thickness variations up to 28%). This intrinsic robustness is a direct consequence of the PNZ material dispersion rather than precise geometric interference. Beyond the specific demonstration in the microwave regime, this work establishes a general theoretical framework for manipulating magnetic light–matter interactions at the subwavelength scale. The concept of gradient zero‐index magnetic metastructures can be readily extended to higher frequency bands (e.g., terahertz or mid‐infrared using antiferromagnetic nanomaterials). This opens new avenues for designing robust nanophotonic devices for nonreciprocal thermal emission management, directional energy harvesting, and integrated optical signal isolation.

## Author Contributions

**Hai‐Feng Zhang:** conceptualization, funding acquisition, project administration, writing – review and editing, supervision. **Junyang Sui:** writing – original draft, investigation, validation, formal analysis, software, visualization.

## Funding

This work was supported by the Open Research Program in China's State Key Laboratory of Millimeter Waves (Grant No. KN202502‐15).

## Conflicts of Interest

The authors declare no conflicts of interest.

## Supporting information


Supporting Information S1

## Data Availability

Research data are not shared.
