# Supplementary material for: Broadband and Wide‐Angle Nonreciprocal Electromagnetic Transport via Gradient Permeability‐Near‐Zero Metastructures
Source: Nanophotonics. 2026 Apr 30;15(9):e70112. doi: 10.1002/nap2.70112 (PMC13642529; doi:10.1002/nap2.70112)
Supplement: Supplementary file 1 [file NAP2-15-e70112-s001.docx]

Supplementary Material

Broadband and Wide-Angle Nonreciprocal Electromagnetic Transport via Gradient Permeability-Near-Zero Metastructures

Junyang Sui^1^, and Hai-Feng Zhang^1,2^*

J. Y. Sui, H. F. Zhang

^1^College of Electronic and Optical Engineering & College of Flexible Electronics (Future Technology),, Nanjing University of Posts and Telecommunications, Nanjing, 210023 ,China

^2^State Key Laboratory of Millimeter Waves, Southeast University, Nanjing, 210096, China

*Corresponding author: hanlor@163.com or hanlor@njupt.edu.cn

1. Optical Properties Calculations of Yttrium Iron Garnet

As described in the main text, when an external magnetic field *H* is applied along the +*y*-axis, the permeability tensor of Yttrium iron garnet (YIG) is expressed as $\overrightarrow{\mu}(\widehat{y}H)=[\mu_{r},0,-i\mu_{k};\mu_{0},1,0;i\mu_{k},0,\mu_{r}]$. In this case, the diagonal and off-diagonal elements are represented as^[1,2]^

$\mu_{r}=1+\frac{\omega_{m}(\omega_{0}-j\Gamma\omega)}{(\omega_{0}-j\Gamma\omega)^{2}-\omega^{2}}$ (S1)

$\mu_{k}=\frac{\omega_{m}\omega}{(\omega_{0}-j\Gamma\omega)^{2}-\omega^{2}}$ (S2)

Here, *j* denotes a unit imaginary number。*ω* and *Γ* are angular frequency and damping constant. *ω*_0_=2π*f*_0_ is the resonance frequency, where *f*_0_=2.8×10^6^×*H_p_* (*p*=1 or 2). *ω_m_*=2π*f_m_* stands for the circular frequency, and *f_m_*=2.8×106×*M_S_*_,_*_p_*. *M_S_*,*_p_* is the saturation magnetization^[1,2]^. According to Maxwell’s equations, the equivalent permeability in the transverse electric (TE) mode and transverse magnetic (TM) mode are given by *μ*_TE_=(*μ_r_*^2^+(*jμ_k_*)^2^)/*μ_r_* and *μ*_TM_=*μ*_0_. Its equivalent refractive index in TE mode is, *ε_f_*=15 is the dielectric constant of YIG^[1,2]^.

2. Dispersion Derivation of Magneto-Optical Structure

**Figure S1.** Schematic of multilayer magnetized materials. The external magnetic field *H* is applied along the +*y*-axis direction.

We investigate the dispersion properties of TE surface modes propagating in a nonreciprocal four-layer stratified structure (see in Figure 2 of main text). The system is defined in a Cartesian coordinate system (*x*,*y*,*z*) where the layers are stacked along the +*z*-direction. The electromagnetic (EM) wave propagates in the *xoz* plane with a propagation constant *β* along the *x*-axis (i.e., fields vary as exp(*j*(*ωt*-*βx*))).

As presentation in **Figure S1**, The structure consists of the following regions (from bottom to top):

(1) Region 0 (Substrate, *z*≤0): Gold (Au). In the microwave regimes, we model the substrate as a Perfect Electric Conductor (PEC) to simplify the boundary conditions (*E*_tan_|*_z_*_=0_=0).
(2) Region 1 (Dielectric, 0<*z*≤*t*_d_): An isotropic dielectric spacer with relative permittivity εd and thickness *t*_d_.
(3) Region 2 (YIG_2_, *t*_d_<*z*≤*z*_2_): The second magneto-optical YIG layer with thickness *t*_2_ (where *z*_2_=*t*_d_+*t*_2_).
(4) Region 3 (YIG_1_, *z*_2_<*z*≤*z*_top_): The first magneto-optical YIG layer with thickness *t*_1_ (where *z*_top_=*t*_d_+*t*_2_+*t*_1_).
(5) Region 4 (Air, *z*>*z*_top_): Free space with permittivity *ε*_0_ and permeability *μ*_0_.

An external static magnetic field is applied in the Voigt configuration, $H=H\widehat{y}$. For the YIG layers (Regions 2 and 3), the relative permeability tensors are $\overrightarrow{\mu_{\text{YIG}1}}(\widehat{y}H)=[\mu_{r1},0,-i\mu_{k1};\mu_{0},1,0;i\mu_{k1},0,\mu_{r1}]$ and $\overrightarrow{\mu_{\text{YIG}2}}(\widehat{y}H)=[\mu_{r2},0,-i\mu_{k2};\mu_{0},1,0;i\mu_{k2},0,\mu_{r2}]$.

Crucially, we define the effective permeability determinant parameter *M* as *M*=*μ_r_*^2^-*μ_k_*^2^.

For TE polarization, the electric field is purely transverse to the propagation plane $E=Ey(z)e^{-j\beta}\widehat{y}$. Starting from Maxwell's curl equation $\nabla\times E=-j\omega\mu_{0}\overline{\mu_{r}}H$, the tangential magnetic field component *H_x_* can be expressed in terms of *E_y_*

$H_{x}=\frac{1}{\omega\mu_{0}M}\left(j\mu_{r}\frac{\partial E_{y}}{\partial z}+\beta\mu_{k}E_{y}\right)$ (S3)

Substituting this into $\nabla\times H=j\omega\varepsilon_{0}\varepsilon_{f}E$ yields the scalar wave equation for the YIG regions

$\frac{\partial^{2}E_{y}}{\partial z^{2}}+\kappa_{f,p}E_{y}=0$ (S4)

Here *p*=1 or 2. Where the transverse wavenumber *κ_f_*_,_*_p_* is defined as

$\kappa_{f,p}=\sqrt{k_{0}^{2}\varepsilon_{f,p}\frac{M_{p}}{\mu_{r,p}}-\beta^{2}}$ (S5)

To derive the dispersion relation efficiently for this multilayer structure, we employ the impedance transfer method. We define the wave impedance (or admittance formulation) at the interface *z* as the ratio of the tangential fields. For convenience, we define a reduced admittance function Ρ(*z*)

$P(z)\equiv\frac{\omega\mu_{0}H_{x}(z)}{jE_{y}(z)}$ (S6)

Using Eq. (S3), the general expression for *Ρ* inside a YIG layer is

$P_{\text{ferrite}}(z)\equiv\frac{1}{M}\left(\mu_{r}\frac{1}{E_{y}}\frac{\partial E_{y}}{\partial z}-j\beta\mu_{k}\right)$ (S7)

(1) Region 1 (Dielectric, 0<*z*≤*t*_d_)

The boundary condition at the PEC substrate (*z*=0) requires *E_y_*(0)=0. Thus, the field takes the form

$E_{y,1}(z)=A_{1}\sin(\kappa_{d}z)$ (S8)

Here, *κ*_d_=(*k*_0_^2^*ε*_d_-*β*^2^)^1/2^. The reduced admittance at the top of the dielectric layer (*z*=*t*_d_) is

$P_{1}(t_{\text{d}})=\frac{1}{E_{y}}\frac{\partial E_{y}}{\partial z}\text{|}_{\text{z=t}_{\text{d}}}=\kappa_{\text{d}}\cot(\kappa_{\text{d}}t_{\text{d}})$ (S9)

(2) Region 2 (YIG_2_, *t*_d_<*z*≤*z*_2_)

We apply the continuity of tangential fields at *z*=*t*_d_, which implies Ρ_2_(*t*_d_^+^)=Ρ_2_(*t*_d_^-^). Let the general solution in YIG_2_ be *E_y_*_,2_(z)=A_2_cos[*κ_f_*_2_(*z*-*t*_d_)]+*B*_2_sin[*κ_f_*_2_(*z*-*t*_d_)]. From Eq. (S7) and the continuity condition, we can derive the admittance looking into the top of YIG_2_ (*z*=*z*_2_=*t*_d_+*t*_2_). Using the transfer matrix property, the explicit recursive relation for the admittance transfer is

$P_{2}(z_{2})=\frac{1}{M_{2}}\left(\mu_{r2}\kappa_{f2}\frac{\Psi_{2}\cos(\kappa_{f2}t_{2})-\sin(\kappa_{f2}t_{2})}{\Psi_{2}\sin(\kappa_{f2}t_{2})+\cos(\kappa_{f2}t_{2})}-j\beta\mu_{k2}\right)$ (S10)

Where Ψ_2_ carries the information from the dielectric layer

$\Psi_{2}=\frac{\mu_{r2}\kappa f_{2}}{M_{2}P_{1}(t_{\text{d}})+j\beta\mu_{k2}}$ (S11)

(3) Region 3 (YIG_1_, *z*_2_<*z*≤*z*top)

Similarly, continuity at *z*=*z*_2_ requires the admittance at the bottom of YIG_1_ to match Ρ_2_(*z*_2_). We define the admittance looking into the top surface of the entire stack (*z*=*z*_top_)

 (S12)

Where the recursive parameter Ψ_3_ is

$\Psi_{3}=\frac{\mu_{r1}\kappa f_{1}}{M_{1}P_{2}(z_{2})+j\beta\mu_{k1}}$ (S13)

(4) Region 4 (Air, *z*>*z*_top_)

The field in the air region must decay exponentially *_Ey_*_,4_∝exp(-*γ*_0_(*z*-*z*_top_)), where *γ*_0_=(*β*^2^-*k*_0_^2^)^1/2^. There reduced admittance of the air region is

$P_{\text{air}}=\frac{\partial E_{y}/\partial z}{E_{y}}=-\gamma_{0}$ (S14)

The dispersion relation of the guided mode is obtained by matching the admittance at the top interface

 (S15)

Substituting Eq. (S12) and Eq. (S14) and rearranging terms, we arrive at the exact transcendental dispersion equation.

$\tan(\kappa f_{1}t_{1}+\delta)=\frac{\mu_{r1}\kappa f_{1}}{j\beta\mu_{k1}-M_{1}\gamma_{0}}$ (S16)

Here, *δ* is the phase shift induced by the underlying YIG_2_ and dielectric layers, defined by

$\tan(\delta)=\Psi_{3}^{-1}=\frac{M_{1}P_{2}(z_{2})+j\beta\mu_{k1}}{\mu_{r1}\kappa_{f1}}$ (S17)

With Ρ_2_(*z*_2_) determined recursively via Eq. (S9) and Eq. (S10).

To validate the physical consistency of our derivation, we consider the limiting case where the thicknesses of the YIG_2_ and dielectric layers vanish (*t*_2_→0, *t*_d_→0).

In this limit:

①Ρ_1_(*t*_d_)→∞ (due to cot(0)), implying a PEC boundary condition.

②Consequently, Ψ_2_→0 and Ρ_2_(*z*_2_)→∞.

③This leads to Ψ_3_→0 (or *δ*→π/2), reducing the tangent term.

Specifically, for a single Ferrite layer directly on PEC (Air/YIG_1_/ PEC), Eq. (S16) simplifies to

$\tan(\kappa f_{1}t_{1})=\frac{\mu_{r1}\kappa_{f1}}{j\beta\mu_{k1}-M_{1}\gamma_{0}}$ (S18)

Eq. (S18) clearly explicitly exhibits non-reciprocal behavior. The term jβμk1 in the denominator changes sign when the propagation direction is reversed (*β*→-*β*). Since *μ_k_*_1_≠0 under an external magnetic field, the dispersion condition differs for forward and backward propagation, i.e., *ω*(*β*)≠*ω*(*-β*), verifying the theoretical basis for the proposed non-reciprocal absorber^[3,4,5]^.

3. Transfer Matrix Method

For the YIG layer, under TE polarization, the electric and magnetic field can be presented as $E=[0,E_{y},0]e^{-j\omega t}$, $E=[H_{x},0,H_{z}]e^{-j\omega t}$, respectively. Based on Maxwell’s equation, an equation for *E_y_* is attained^[1,2]^

$\frac{\partial^{2}E_{y}}{\partial x^{2}}+\frac{\partial^{2}E_{y}}{\partial z^{2}}+k_{\text{YIG}}^{2}E_{y}=0$ (S19)

 (S20)

Based on the boundary conditions, the transfer matrix can be deduced^[1,2]^

$M_{\text{YIG}}=\left[\begin{matrix} \cos(k_{\text{YIG}z}d_{p})-\frac{k_{\text{YIG}x}\mu_{k}}{k_{\text{YIG}z}\mu_{r}}\sin(k_{\text{YIG}z}d_{p}) & j\eta_{p}\sin(k_{\text{YIG}z}d_{p}) \\ \frac{j}{\eta_{p}}\left(1+\left(\frac{k_{\text{YIG}x}\mu_{k}}{k_{\text{YIG}z}\mu_{r}}\right)^{2}\right)\sin(k_{\text{YIG}z}d_{p}) & \cos(k_{\text{YIG}z}d_{p})+\frac{k_{\text{YIG}x}\mu_{k}}{k_{\text{YIG}z}\mu_{r}}\sin(k_{\text{YIG}z}d_{p}) \end{matrix}\right]$ (S21)

where the wave vectors along the z-axis direction and y-axis direction are *k*_YIG_*_z_*=*k*_YIG_cos*θ*, *k*_YIG_*_x_*=k_YIG_sin*θ*. $\eta_{p}=\sqrt{\frac{\mu_{0}}{\varepsilon_{0}}}n_{\text{YIG}}/\cos\theta$, where *p*=1 or 2.

As for conventional dielectric layers, the transfer matrix is^[6]^

$M_{\text{YIG}}=\left[\begin{matrix} \cos(k_{z}d) & \frac{j\sin(k_{z}d)}{\eta} \\ j\eta\sin(k_{z}d) & \cos(k_{z}d) \end{matrix}\right]$ (S22)

where *k_z_* and *η* are the relevant optical property of isotropic dielectric layer.

When the EM wave passes through the proposed structure in Figure 2, the entire transfer matrix, which connected the input and output EM field can be deduced by analogy^[6]^

$\left[\begin{matrix} E_{1} \\ H_{1} \end{matrix}\right]=M_{\text{YIG1}}M_{\text{YIG2}}M_{\text{Al2O3}}M_{\text{AU}}\left[\begin{matrix} E_{N+1} \\ H_{N+1} \end{matrix}\right]=\left[\begin{matrix} m_{11} & m_{12} \\ m_{21} & m_{22} \end{matrix}\right]\left[\begin{matrix} E_{N+1} \\ H_{N+1} \end{matrix}\right]$ (S23)

The reflection coefficient *r*(*f*,*θ*,*φ*) and transmission coefficient *t*(*f*,*θ*,*φ*) are as follows^[6]^

 (S24)

 (S25)

where *η*_0_=*η_N_*_+1_=(*ε*_0_/*μ*_0_)cos*θn*_air_ (*n*_air_=1) under the condition of background medium is all air. Then, the reflectivity *R*(*f*, *θ* ,*φ*), transmissivity *T*(*f*, *θ*, *φ*) and absorptivity *α*(*f*, *θ*, *φ*) can be gained^[6]^

 (S26)

 (S27)

 (S28)

4. Feasible Experimental Process for the Nonreciprocal Metastructure

Although this work focuses on theoretical innovation, the proposed gradient Permeability-Near-Zero (PNZ) metastructures are highly feasible for experimental realization using mature microwave engineering techniques. A feasible experimental plan is outlined below.

For the fabrication process: i) YIG layers growth. The top ferrite layers (YIG_1_ and YIG_2_) can be grown using the Liquid Phase Epitaxy (LPE) method or Radio Frequency (RF) magnetron sputtering^[7]^. The required differences in saturation magnetization *M_S_* and damping constants *Γ* for YIG_1_ and YIG_2_ can be precisely achieved by controlling the doping concentration of Gallium (Ga) or Aluminum (Al) during the epitaxial growth process, which is a well-established and highly controllable technique in modern microwave ferrite engineering^[8,9]^. ii) Dielectric and substrate integration. The intermediate Al_2_O_3_ dielectric spacer can be deposited using Atomic Layer Deposition (ALD) or directly utilized as a commercial low-loss ceramic substrate. The bottom 1 μm thick Gold (Au) layer can be deposited via electron beam evaporation, whose thickness far exceeds the skin depth in the 35~40 GHz regime.

For the experimental measurement setup, the measurement of broadband and wide-angle nonreciprocal absorption requires a customized free-space microwave test bench, similar to configurations successfully deployed in recent gyromagnetic metasurface studies^[10,11]^.

i) Setup details. The fabricated sample will be mounted on a motorized rotational stage located inside a microwave anechoic chamber to eliminate background reflections. Two broadband horn antennas (operating in the *K*-band, 26.5~40 GHz) will be connected to a Vector Network Analyzer (VNA) to serve as the transmitter and receiver.
ii) Magnetic biasing. To excite the PNZ properties, different external magnetic fields must be applied to the adjacent YIG layers. For practical experimental realization, this can be achieved utilizing gradient magnetic field coils integrated around the sample stage. As demonstrated by Xuan *et al*.^[12]^, applying specific currents to gradient coils generates a spatially linear magnetic field. This technique fulfills the requirement of establishing distinct static magnetic field intensities (up to *H*_1_=12000 Oe, *H*_2_=12900 Oe) parallel to the +*y*-axis at different YIG layers while effectively isolating the influence of adjacent magnetic fields.

iii) Data acquisition. By rotating the sample stage to specific incidence angles (+*θ* and -*θ*), the VNA will record the reflection parameters (*S*_11_ and *S*_21_). The absorptivity can then be experimentally extracted using *α*=1-|*S*_11_|^2^-|*S*_21_|^2^ (where |*S*_21_|^2^→0 due to the opaque Au substrate), directly validating the asymmetric absorption spectra generated by our numerical simulations.

5. Artificial Rabbits Optimization: A New Bio-Inspired Meta-Heuristic Algorithm for Solving Engineering Optimization Problems

The structural and material parameters of the proposed gradient PNZ metastructures encompass a high-dimensional continuous search space. To efficiently navigate this space and locate the global optimum for broadband nonreciprocal absorption, we employed the Artificial Rabbits Optimization (ARO), a robust bio-inspired meta-heuristic algorithm highly suitable for solving complex engineering optimization problems^[13,14]^.

The explicit optimization workflow, illustrated in Figure S2a, requires defining the optimization objective, the parameter space, and the algorithmic strategies. The ultimate goal of the metastructure is to strictly satisfy the broadband nonreciprocal bounds within the 35~40 GHz frequency band, requiring high absorption in the forward direction (*α*(*λ*,*θ*,*φ*)>0.9) and low absorption in the backward direction (*α*(*λ*,-*θ*,*φ*)<0.1). To enforce these specific threshold criteria, we formulated a penalty-based fitness function *F*(*x*). The algorithm is designed to minimize *F*(*x*), which calculates the cumulative constraint violations across the target spectrum:

 (S29)

where *f_i_* represents the discrete frequency points sampled uniformly across the 35~40 GHz range, and *N* is the total number of sampling points. In this formulation, if a structural parameter set perfectly meets the criteria at a specific frequency point, its local penalty drops to exactly zero. The algorithm will continue optimizing until *F*(*x*) approaches zero, ensuring the robust nonreciprocal target is fully achieved. The optimization vector *x* is defined by the physical constraints of the materials and the fabrication feasibility. The components of *x* and their respective search boundaries are explicitly defined as follows. i) Thickness of the YIG layers: 0 μm≤*d*_YIG1_,*d*_YIG2_≤500 μm; ii) Thickness of the dielectric spacer: 0 μm≤*d*_Al2O3_≤500 μm; iii) External magnetic field: 12000 Oe≤*H*_1_,*H*_2_≤13000 Oe; iv) YIG damping constants: 0.001≤*Γ*_1_,*Γ*_2_≤0.025; v) Saturation magnetizations: 100 Oe≤*M_S_*_1_,*M_S_*_2_≤1800 Oe. The ARO algorithm initializes by randomly generating a population of 30 “rabbits” (candidate parameter sets) within the defined boundaries. Using the TMM, the nonreciprocal spectra for each parameter combination are computed to evaluate their initial fitness values. The algorithm then orchestrates the search via an energy factor *A*(*t*), which dynamically regulates the transition between global exploration and local exploitation:

i) Exploration phase (detour foraging). When *A*(*t*)>1, the rabbits adopt a detour foraging strategy, perturbing their positions towards randomly selected peers. This mechanism prevents the algorithm from trapping into local optima and ensures a thorough exploration of the broad parameter space.

ii) Exploitation phase (random hiding). When *A*(*t*)≤1, the rabbits shift to the random hiding strategy, refining their positions within the local vicinity of the best-known solution to fine-tune the high-performance structural parameters^[15]^.

Throughout the iterations, the algorithm strictly updates the positions if the new fitness is lower than the current one. The optimization terminates when it reaches the predefined maximum iterations (*t*_max_=2000) or strictly satisfies the robust nonreciprocal requirements (*α*(*λ*,*θ*,*φ*)>0.9 and *α*(*λ*,-*θ*,*φ*)<0.1). Figure S2b illustrates the relationship between each iteration and the average fitness, with the fitness smoothly and rapidly decreasing from 162 and reaching a minimum value of 3.01 at *It*=731, indicating that the solution *d*’ is optimal for achieving the target response.

**Figure S2.** (a) The flowchart of the Artificial Rabbits Optimization algorithm. (b) Evolution of the algorithm for the optimization target responses (*α*(*λ*,*θ*,*φ*)>0.9 and *α*(*λ*,-*θ*,*φ*)<0.1 at 35~40 GHz), showing the rapid convergence relationship between the average fitness and the number of iterations.

**6. The verification of the correctness of the simulation results**

**Figure S3.** The spectrum simulated by the TMM (solid curves) and the FIM (dotted curves) corresponding to the metastructures.

To validate the accuracy of the simulated structure, we employed the Transfer Matrix Method (TMM)^[6]^ and the Finite Integration Method (FIM)^[16]^ to simulate the spectral response of metastructures. Corresponding to Figure 3a in the manuscript, Figure S3a presents the spectra of the broadband nonreciprocal absorptivity under *θ*=±72° incident angle, simulated using TMM and FIM. The results indicate a close agreement between the spectra produced by both algorithms, both in terms of values and trends. The subtle differences at specific frequent points can be attributed to that when the 72° incident angle of the electromagnetic waves, since TMM is an analytical solution method and FIM is a numerical solution method, truncation errors can arise during the meshing process^[17,18]^. Additionally, there are inherent differences between the two algorithms in handling boundary conditions, phase, and numerical errors^[17,18]^. However, these algorithmic discrepancies have minimal impact on the overall performance of the structure and do not affect the corresponding conclusions.

**Figure S4.** Absorptivity spectra at *θ*=±72° under TM mode.

Figure S4 shows the absorption rates of the metastructure at positive and negative incident angles under the same incident conditions as in Figure 3a. Since the magnetic permeability tensor of YIG is equivalent to *μ*_0_ for the TM mode and cannot break time-reversal symmetry, no non-reciprocal absorption occurs. Furthermore, since the dielectric constant of YIG has no imaginary component and the imaginary component of the dielectric layer in the metastructure is also nearly zero, the multilayer film exhibits high reflectivity when simulated on a gold substrate. This ultimately results in *α*(*f*,72°,0°) and *α*(*f*,-72°,0°) being equal at an absorption rate of the order of 1×10^-3^, as shown in Figure S4.

**7. Influence of Gradient Magnetic Field on Bandwidth**

**Figure S5.** Performance and analysis of the same external magnetic field on metastructures. Under the *H*_1_ and *H*_2_ are equal to 12000 Oe, (a) the permeability tensor curves for YIG_1_ and YIG_2_, and (b) the nonreciprocal absorptivity spectra at *θ*=±72°. Under the *H*_1_ and *H*_2_ are equal to 12900 Oe, (a) the permeability tensor curves for YIG_1_ and YIG_2_, and (b) the nonreciprocal absorptivity spectra at *θ*=±72°.

To elucidate the crucial role of applying different external magnetic fields (*H*_1_≠*H*_2_) to the YIG1 and YIG2 layers, we conduct a comparative analysis by simulating the metastructure under the same external magnetic field (e.g. *H*_1_=*H*_2_=12000 Oe or *H*_1_=*H*_2_=12900 Oe).

As shown in Figure S5, when the two YIG layers are subjected to the identical magnetic field, their PNZ frequencies shift close to each other. Because the PNZ point is highly sensitive to the external magnetic field strength, the lack of a magnetic field gradient causes the transverse electric-polarized leaky modes (TPLM) of both layers to be excited at nearly the same frequency.

Consequently, the nonreciprocal absorption peaks severely overlap, leading to high nonreciprocity only within a very narrow frequency band. This configuration drastically weakens the broadband performance of the metastructure. Therefore, the introduction of a spatial linear magnetic field (gradient magnetic field) is not only experimentally feasible via gradient coils but also physically indispensable for staggering the PNZ frequencies and achieving ultra-broadband nonreciprocal absorption across the 35~40 GHz range.

**8. Sensitivity Analysis of Intrinsic Material Parameters and Dielectric Loss on Non-reciprocal Contrast**

**Figure S6.** Sensitivity analysis illustrating the decay of the average non-reciprocity as a function of (a) the damping coefficient error Δ*Γ*, and (b) the saturation magnetization error Δ*M_S_*.

In practical microwave engineering, the fabrication of YIG thin films via processes such as LPE inevitably introduces minor material imperfections. Factors including lattice mismatch, microscopic defects, or slight deviations in doping concentrations can cause the intrinsic magnetic properties, specifically the damping coefficient *Γ* and saturation magnetization *M_S_*, to drift from their ideal designed values.

To quantitatively validate the intrinsic robustness of the proposed gradient PNZ metastructure against such practical manufacturing uncertainties, we conducted a rigorous sensitivity analysis on these intrinsic material parameters. We systematically introduced error percentages to the optimal design values of both *Γ* and *M_S_* and calculate the resulting average non-reciprocity across the target broadband spectrum.

Although the TPLM mechanism couples with magnetic loss, the system is remarkably insensitive to deviations in *Γ*. Figure S6a shows that the metastructure sustains an average non-reciprocity strictly greater than 0.6 across an exceptionally broad *Γ* error range from -67% to 422%. This immense tolerance window indicates that even severe lattice defects during LPE growth will not critically compromise the non-reciprocal absorption.

Fluctuations in *M_S_* inherently shift the localized PNZ resonance frequencies of the respective YIG layers. However, owing to the ultra-broadband nature of the engineered cavity resonance, these localized frequency shifts are effectively buffered. The average non-reciprocity robustly remains above 0.6 even when the *M_S_* error ranges from -52% to +73%, as shown in Figure S6b. These massive tolerance thresholds physically guarantee that the inverse-designed gradient metastructure possesses exceptional intrinsic robustness, making it highly reliable for practical fabrication despite inevitable material parameter drifts.

**Figure S7.** Sensitivity analysis illustrating the decay of the average non-reciprocity as a function of the dielectric loss tangent of the Al_2_O_3_ spacer.

In practical material preparation and micro/nanofabrication, dielectrics inevitably exhibit a complex permittivity *ε*=*ε*’-*iε*’’, where the imaginary component characterizes the dielectric loss, commonly quantified by the loss tangent (tan*δ*=*ε*’’/*ε*’). For resonant metastructures, small-angle absorption is significantly enhanced through multiple wave reflections within the optical cavity. Consequently, the cumulative attenuation induced by dielectric loss could potentially dampen the cavity resonance and weaken the overall non-reciprocity *η*_average_.

Although previous studies have established that the intrinsic loss tangent of Al_2_O_3_ is practically negligible (typically <10^-4^) in the 35~40 GHz microwave band^[19]^, we conducted a rigorous sensitivity analysis to investigate the operational boundaries of our design under sub-optimal fabrication scenarios. We artificially introduced an increasing loss tangent to the Al_2_O_3_ spacer and evaluated the decay of the average non-reciprocity. As illustrated in Figure S7, the average non-reciprocity gradually decays as the dielectric loss increases, which physically aligns with the weakening of the cavity-enhanced TPLM effect due to internal energy dissipation.

Nevertheless, the metastructure exhibits extraordinary intrinsic robustness. For the optimal designed cavity thickness of 480 μm, the average non-reciprocity remains highly effective, sustaining a value greater than 0.6 as long as the loss tangent is maintained within the broad range of 0~0.0146. Since the acceptable threshold (0.0146) is exponentially higher than the actual material loss of Al_2_O_3_ in the target spectrum, we can conclude that the proposed gradient PNZ metastructure will reliably maintain a high non-reciprocal contrast in realistic engineering applications, largely immune to typical dielectric loss induced by fabrication variations.

**References**

1．C. Liang, M. Wu, F. Li, S. Li, and Y. Poo, "Diffusive metasurfaces based on transverse magnetized ferrite for reduction of radar cross section," Opt. Express 32, no. 11 (May 2024): 18972–18983.
2．Y.-R. Wu, R.-Y. Dong, J. Xu, and H.-F. Zhang, "A novel CPA-based layered photonic structure for multipurpose sensing applications," Opt. Laser Technol. 163 (August 2023): 109422.
3．S. R. Seshadri, "Surface Magnetostatic Modes of a Ferrite Slab," Proc. IEEE 58, no. 3 (March 1970): 506–507.
4．R. W. Damon and J. R. Eshbach, "Magnetostatic modes of a ferromagnet slab," J. Phys. Chem. Solids 19, no. 3-4 (May 1961): 308–320.
5．L. Zhu and S. Fan, "Near-field photonic Hall effect and its applications in nonreciprocal photon transport," Phys. Rev. B 90, no. 22 (December 2014): 220301.
6．L. Qi, Z. Yang, F. Lan, X. Gao, and Z. Shi, "Properties of surface magnetostatic waves in a sandwich structure with a plasma layer," Phys. Plasmas 17, no. 4 (April 2010): 042501.
7．C. Kshirsagar, R. Bidthanapally, Y. Liu, et al., “Seed-Layer-Assisted Liquid-Phase Epitaxial Growth of YIG Films on Single-Crystal Yttrium Aluminum Garnet Substrates: Evidence for Enhancement in Strain-Induced Anisotropy,” Crystals 15, no. 11 (2025): 953.
8．V. G. Harris. “Modern microwave ferrites,” IEEE Transactions on Magnetics 48, no. 3 (2011): 1075-1104.
9．M. C. Onbasli, A. Kehlberger, D. H. Kim, et al., “Pulsed laser deposition of epitaxial yttrium iron garnet films with low Gilbert damping and bulk-like magnetization,” Apl Materials 2, no. 10 (2014).
10．Y. Zhang, L. Jing, C. Niu, et al. “Broadband nonreciprocal gyromagnetic metasurface via magnetic Kerker-type dimers,” Applied Physics Letters 124, no. 23 (2024).
11．W. Yang, J. Qin, J. Long, et al. “A self-biased non-reciprocal magnetic metasurface for bidirectional phase modulation,” Nature Electronics 6, no. 3 (2023): 225-234.
12．L. Xuan, X. Kong, J. Wu, et al. “A smoothly-connected crescent transverse gradient coil design for 50mT MRI system,” Applied Magnetic Resonance 52, no. 6 (2021): 649-660.
13．L. Wang, Q. Cao, Z. Zhang, S. Mirjalili, and W. Zhao, "Artificial Rabbits Optimization: A New Bio-Inspired Meta-Heuristic Algorithm for Solving Engineering Optimization Problems," Eng. Appl. Artif. Intell. 114 (October 2022): 105082.
14．R. Wang and S. Zhang, "A new physics-informed neural network for solving electromagnetic inverse scattering problems," Sci. Rep. 14 (August 2024): 18295.
15．F. Anka, N. Agaoglu, S. Nematzadeh, M. Torkamanian-Afshar, and F. S. Gharehchopogh, "Advances in Artificial Rabbits Optimization: A Comprehensive Review," Arch. Comput. Methods Eng. 32, no. 4 (January 2025): 2113–2148.
16．M. Clemens and T. Weiland. “Discrete electromagnetism with the finite integration technique,” Progress In Electromagnetics Research 32 (2001): 65.
17．P. H. Wen, Y. C. Hon, M. Li, et al. “Finite integration method for partial differential equations,” Applied Mathematical Modelling 37, no. 24 (2013): 10092-10106.
18．B. Rahman and J. Davies. “Finite-element solution of integrated optical waveguides,” Journal of Lightwave Technology 2, no. 5 (2003): 682-688.
19．L. V. Rodriguez de Marcos, M. Christophersen, M. F. Batkis, J. G. del Hoyo, J. Patel, E. J. Wollack, J. A. Christodoulides, J. I. Larruquert, M. A. Quijada, “Self-consistent optical properties of PEALD-Al2O3 thin films,” Opt. Materials Express 7 (2016) 1750-1764.
